# The Anti-melanogenesis Activities of Some Selected Red Macroalgae from Northern Coasts of the Persian Gulf

**Published:** 2019

**Authors:** Foroogh Namjoyan, Massoumeh Farasat, Mojtaba Alishahi, Alireza Jahangiri, Hamideh Mousavi

**Affiliations:** a *Marine Pharmaceutical Research Center, School of Pharmacy, Ahvaz Jundishapur University of Medical Sciences, Iran.*; b *Department of Biology, School of Sciences, Ahvaz Branch, Islamic Azad University, Ahvaz, Iran.*; c *Department of Clinical Sciences, School of Veterinary Medicine, Shahid Chamran University, Ahvaz, Iran.*; d *Department of Medicinal Chemistry, School of Pharmacy, Ahvaz Jundishapur University of Medical Sciences, Ahvaz, Iran. *; e *Department of Pharmacognosy, School of Pharmacy, Ahvaz Jundishapur University of Medical Sciences, Iran.*

**Keywords:** Algae, Persian Gulf, Melanogenesis, Mushroom tyrosinase, Zebrafish

## Abstract

Tyrosinase is a key enzyme in melanin production. Therefore, tyrosinase inhibitors are used in cosmetic and medicinal industries to prevent or treat overproduction of melanin such as melasma, solar lentigo and post inflammatory melanoderma. Due to safety of natural whitening agents, in the present study, *in-vitro* anti-tyrosinase and *in-vivo* anti-melanogenesis activities of some selected red macroalgae of the Persian Gulf were investigated. The effects of various concentrations (100, 250 and 500 µg/mL) of methanolic extracts of three red macroalgae including *Digenea simplex (D. simplex),* *Laurencia papillosa,* and *Laurencia paniculata* on the activity of diphenolase of mushroom tyrosinase were studied by using L-Dopa as substrate. Subsequently, the activity of macroalgae with high inhibitory effect on hydroxylation of L-tyrosine was investigated by mushroom tyrosinase and zebrafish model. Anti-melanogenesis effects of algae extracts were studied on zebrafish as an alternative *in-vivo* model. Kojic acid was used as a positive control. All the tested macroalgae showed significantly a lower inhibitory effect on activities of diphenolase and monophenolase (of mushroom tyrosinase) compared to kojic acid.* D. simplex* showed the most anti-tyrosinase activity in zebrafish model among the samples. *D. simplex* extract and Kojic acid inhibited tyrosinase activity by 43.18% and 50.45%, and decreased total melanin content of zebrafish by 47.27% and 50.21%, respectively.

## Introduction

Melanin pigment is widely distributed in bacteria, fungi, plants, and animals and is produced through melanogenesis process. Melanin content of mammals is the main determining factor of the skin and hair colors (1). In addition, melanin protects the skin against the harmful effects of ultraviolet radiation and reactive oxygen species (ROS) (2). Tyrosinase (EC 1.14.18.1) is a multifunctional and rate-limiting enzyme in melanin biosynthetic pathway which is involved in two distinct reactions. Firstly, it catalyses hydroxylation of L-tyrosine to L-Dopa (monophenolase activity) and secondly, converts L-Dopa to Dopaquinone by oxidation (diphenolase activity) (2, 3). Also, tyrosinase is responsible for undesired browning of fruits, vegetables, and reduces nutritional values and impairs the color and sensory properties such as flavor and texture (softening) (4). Tyrosinase inhibitors could be used in pharmaceutical and cosmetic industries for prevention and treatment of melanin overproduction-mediated diseases such as melasma, solar lentigines, and ephelides. Besides, tyrosinase inhibitors could prevent enzymatic browning in fruits, vegetables, and seafoods and could be used in food industries (1, 2). There are several mechanisms to melanogenesis inhibition. Tyrosinase inhibition is the most common way to achieve reduction of pigment in the skin (5). Available inhibitors of tyrosinase such as hydroquinone and kojic acid have toxicity and low effectiveness. So, researchers are seeking for safer and more effective inhibitors. Hence, naturally occurring compounds are becoming increasingly important in the development of tyrosinase inhibitors (2, 6). Seaweeds are rich in various biochemical compounds and are good choices to explore new biological metabolites with a wide diversity of physiological and biochemical activities (7, 8).

The skin care effects such as whitening, sun protection, antioxidant, antibacterial, antifungal, moisturizing, anti-aging, and anti-wrinkle were reported from macroalgae (8). Due to suitable environmental conditions of coastal waters of Iran, there is a great potential for the discovery of lead compounds that could be used in pharmaceutical industry (9). Apparently, there is no study has done on anti-melanogenesis of macroalgae from south of Iran. However, the aim of this study is to investigate the whitening effect of red macroalgae using cell free mushroom tyrosinase (*In-vitro*) and zebrafish model (*In-vivo*). 

## Experimental

*Chemicals*

Mushroom tyrosinase (3130 unit/mg), L-DOPA (powder, ≥98.0%), L-tyrosine (powder, ≥98.0%), Synthetic melanin, DMSO (Dimethylsulfoxide), and methanol were purchased from Sigma Chemical Co. (St. louis, MO, USA). Pro-prep and Pro-measure protein solutions were purchased from Intron Biotechnology (Korea). Kojic acid was obtained from Fluka and the other chemicals used were of analytical grades.

*Macroalgae collection*

The red macroalgae was collected at low tide time from the coastal areas of Jofreh and Bandargah (Bushehr) in December 2010 and January 2012. After harvesting, sands, salts, and epiphytes were removed with fresh water and then, cut into appreciate size and were air-dried at room temperature with good-controlled air condition carefully. The samples were milled into powder and kept at -80 °C for further analysis. The voucher specimens were pressed and stored in 5% formol for identification. Seaweeds were identified by Dr. Massoumeh Farasat using morphological and anatomical examinations of cell structures with the aid of identification keys in the taxonomic publications (10-13). Voucher specimens were deposited in Jundishapour Marine Pharmaceutical Research Center herbarium.

*Algal extracts preparation*

Dried algal powder (200 mg) was extracted with 6 mL 80% methanol in an ultrasonic bath for 30 min, vortexed, and then were kept at room temperature and dark place for 48 h. After one more time vortexing, the extracts were centrifuged at 10000×g for 15 min, filtered through watman No.1 filter paper and then, the extracts were dried and weight of dried extracts were calculated. The dried extracts were dissolved in 3% DMSO and adjusted to final concentrations of 100, 250, and 500 μg/mL. 

*In-vitro: mushroom tyrosinase inhibitory assay*

The inhibitory effects of red macroalgae were investigated by cell-free mushroom tyrosinase assay according to chan *et al*. with some modifications (14). The tyrosinase inhibitory activity was determined useing L-Dopa and L-tyrosine as substrates. In brief, 100 μL of 200 unit/mL of mushroom tyrosinase in 25 mM phosphate buffer (pH 6.8) was added to 50 μL of different concentrations of extracts (100, 250 and 500 μg/mL) in 96-well plate and the absorbance of wells were recorded at 70-sec intervals (10 cycles) at 475 nm with microplate reader (Tecan sunrise, Switzerland). This experiment was done for measuring and correcting the interference of the excess absorbance of the phenolic compounds in the extract. Then, 100 μL of L-Dopa (2.5 mM) or L-tyrosine (1.5 mM) were added to mixture reaction and absorbance was measured at 70-second intervals (20 cycles) at 475 nm. Kojic acid (50, 100, 250 and 500 μg/mL) and 3% DMSO were used as positive and negative controls, respectively. 

Percentage of inhibition of tyrosinase activity was calculated as: 

Inhibition% = {[(A - B) - (C - D)]/(A - B)} × 100 

A: Absorbance of the enzyme, substrate, and DMSO solution. 

B: Absorbance of the substrate and DMSO solution. 

C: Absorbance of the enzyme, substrate and extract solution. 

D: Absorbance of the substrate and extract solution. 

*In-vivo assay*

*Origin and maintenance of parental zebrafish*

Adult zebrafish were obtained from a commercial dealer. Male and female zebrafish were kept in two separate acrylic tanks at 28.5 °C under the light/dark cycle of 14/10 h. Zebrafish were fed 3 times a day, 6 days a week with bloodworm food and supplemented with daphnia. In the evening, 6 fishes in 1: 2 ratio of female: male groups were added to 5 L tanks that have been marbled and filled with fresh water. By turning on the light in the morning, natural spawning was induced. The lucid fertilized eggs were collected for further experiments. 

*Tyrosinase inhibitory activity assay*

Tyrosinase inhibitory effects of red macroalgae were determined according to Choi *et al*. and Cha *et al*. with some modifications (15, 16). In a 6-well plate, 100 synchronized embryos were added to each well containing 6650 μL of sterile fresh water and were kept in incubator at 28.5 °C. After 9 hpf (hours post fertilization), 350 μL of the algal extracts (100 μg/mL) was added. For ensuring the even distribution of the compounds in the wells, replacement of medium was done once a day. After 48 hpf, zebrafish embryos were collected and sonicated in 6 mL pro-prep protein extraction solution in 15 mL falcon tube for extraction of enzyme and melanin. Lysate was cleared by centrifuging at 10000×g for 5 min. Supernatant was aspirated and pellet put aside for determination of melanin content. After quantitation of supernatant by pro-measure kit, the samples were adjusted by pro-prep solution to 250 μg pr/100 μL. Then, 100 μL 1.5 mM L-tyrosine was added to 100 μL of samples in 96-well plate and subsequently was incubated at 28 °C for 60 min. Absorbance of samples was measured at 475 nm. Blank well contained 100 μL of pro-prep solution and 100 μL of 1.5 mM L-tyrosine. The blank absorbance was eliminated from each absorbance value. Kojic acid (100 μg/mL) and 0.1% DMSO were used as positive and negative controls, respectively. The final activity was expressed as a percentage to the negative control. 

Percentage of inhibition of tyrosinase activity was calculated as: 

Inh% = [{(A - B) - (C - B)}/(A - B)] × 100

A: Absorbance of negative control

B: Absorbance of blank

C: Absorbance of samples

*Melanin content of zebrafish embryos*

The pellet was dissolved in 1 mL of 1 N NaOH at 100 °C for 30 min. The mixture was vigorously vortexed to solubilize the melanin pigment. 

The absorbance of the supernatant was measured at 490 nm. NaOH was considered as blank. The results were compared with a standard melanin curve (concentrations 1, 5, 10, 25, 50, 100, 200 and 300 μg/mL). The melanin content was calibrated by protein amount, and expressed as a percentage to negative control. 

Percentage of inhibition of melanin synthesis was calculated as: 

Inh% = [{(A - B) - (C - B)}/(A - B)] × 100

A: Melanin content of negative control

B: Melanin content of blank

C: Melanin content of samples

*Zebrafish pigmentation evaluation*

Six Synchronized embryos were collected and transferred to each well in a 12-well plate containing 1800 μL of sterile water and incubated at 28.5 °C. Two-hundred μL of algal extract (100 μg/mL) was added to embryo medium after 9 hpf. Similarly to tyrosinase inhibitory activity assay, replacement of the medium was done. After 48 hpf, the embryos chorions were removed by forceps, anesthetized in tricaine methansulfonate solution and photographed under the stereomicroscope. 

*Statitical analysis*

Experiments were performed in triplicate and the data were expressed as the mean ± standard error. One-way ANOVA was used to compare the mean values of each treatment. A value of *p* < 0.05 was regarded as statistically significant in all experiments. 

## Results and Discussion

During the study, 3 red macroalga species, including *Laurencia papillosa*, *L. paniculata,* and *Digenea simplex *were collected from the northern coasts of the Persian Gulf. More information on location and collection time of these species are listed in Table 1. According to our data, there are no reports on anti-melanogenic effects of our selected red macroalgae. So, the aim of this study was to investigate the whitening effect of red macroalgae by *in-vitro* and *in-vivo* techniques including cell free mushroom tyrosinase and zebrafish model. 

**Table 1:** Location and collection time of macroalgae

**ID Code**	**Species**	**Location**	**Time of collection**
R100111	*D. simplex*	Bandargah (Bushehr)	December
R121311	*L. papillosa*	Jofreh	January
R121312	*L. paniculata*	Jofreh	January

**Table 2:** Inhibitory effects (%) of macroalgae on mushroom tyrosinase

**Conc (µg/mL)**	***D. simplex***	***L. papillosa***	***L. paniculata***	**Kojic acid**
500	31.89 ± 0.628	17.55 ± 0.126	10.85 ± 0.650	93.54 ± 0.220
250	30.80 ± 0.711	16.68 ± 0.221	9.35 ± 0.298	92.33 ± 0.340
L-Dopa				
100	28.78 ± 0.325	14.82 ± 0.450	8.1 ± 0.308	81.85 ± 0.190
50	Nt	Nt	Nt	78.81 ± 0.310
500	9.98 ± 1.245	Nt	Nt	100
250	2.90 ± 0.935	Nt	Nt	100
L-tyrosine				
100	NI	Nt	Nt	100
50	Nt	Nt	Nt	94.55 ± 0.120

Each value is expressed as the means ± SE (n = 3). Nt: Not tested.

NI: No inhibition.

**Figure 1:**
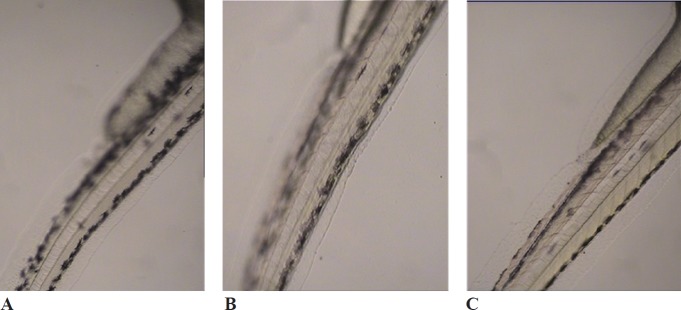
Effects of melanogenic inhibitors on the pigmentation of zebraﬁsh embryos. (A) without inhibitor, (B) *D. simplex *and (C) kojic acid

**Figure 2:**
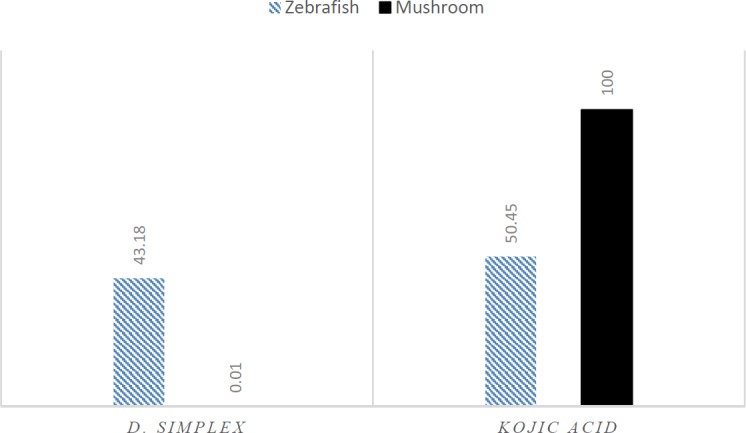
Effects of *D. simplex *and kojic acid on oxidation of L-tyrosine by mushroom and zebrafish tyrosinase at 100 μg/mL

The effects of macroalgae on mushroom tyrosinase were investigated using L-Dopa as a substrate. All the samples showed significantly lower inhibitory effect on tyrosinase compared to kojic acid as positive control (*p* < 0.05) (Table 2). *D. simplex *showed the most anti-tyrosinase activity on oxidation of L-Dopa among samples.

*L. papillosa* showed anti-tyrosinase activity more than *L. paniculata*. Differences between species may be the result of their different biologically active compounds. Many studies have been done to evidence anti-tyrosinase activity of red macroalgae. For instance, *L. okamurae* extract was tested for anti-tyrosinase activity on hydroxylation of L-tyrosine and 27% inhibition at concentration of 100 μg/mL in comparison to kojic acid (97.61%) reported by Cha *et al*. (2011). They stated that anti-tyrosinase active compounds of *L. okamurae* were stable to heat (15).

UV radiations can induce production of reactive oxygen species (ROS) in skin, leading to enhance melanin synthesis, DNA damage, and proliferation of melanocytes. Thus, ROS scavenging compounds and redox agents like antioxidants are effective in the treatment of hyperpigmentation (17). Compounds with reduction potential can have depigmenting effects through two ways: by interacting with *O*-quinones, and suppression of the oxidative polymerization of melanin intermediates or by tyrosinase inhibition through reacting with copper in the active site of the enzyme (18). The molecular models have revealed that the sulfur atom of tyrosinase inhibitors is essential for interaction with the copper ions in the active site of tyrosinase (19). Red seaweeds› galactans are sulfated polysaccharides which are classified as agarans and carrageenans (20). Carrageenans have exhibited strong antioxidant, antibacterial, anti-inflammatory and a broad spectrum of the other therapeutic properties (21). Previous studies have shown antioxidant and free radical scavenging effects of *L. papillosa*. Nahas *et al*. reported that non-polar extracts of *L. papillosa* had more antioxidant activities than its polar extracts, which shows that less polar compounds are responsible to its antioxidant activity (22). There might be a relationship between anti-tyrosinase and antioxidant effects of *L. papillosa* and the presence of sulfated polysaccharides. Other skin care effects like antibacterial, antifungal, wound healing, and anti-herpes simplex virus effects also have been reported in previous studies for *L. papillosa* (23-27).

Due to higher anti-tyrosinase activity of *D. simplex*, it was selected for further experiments. Its effect on monophenolase activity of mushroom tyrosinase was investigated using L-tyrosine as a substrate. Results showed that anti-tyrosinase effect of methanolic extract of *D. simplex* was significantly less than kojic acid (*p* < 0.05) (Table 2). *D. simplex* inhibited diphenolase activity of mushroom tyrosinase more than monophenolase activity that might be related to inhibition mechanism of enzyme. Several studies have shown the antibacterial, antifungal, antioxidant, and anti-inflammatory activities for *D. simplex* (28-31). Besides, anti-tyrosinase activities of some red algae have been already shown. Cha *et al*. (2011) in their study on twenty red macroalgae found that algal extraction temperature had influence on mushroom tyrosinase activity. They reported that by increasing the extraction temperature, the inhibitory effects were increased. The inhibitory activities of algal extracts varied between 9.95 to 90.75% (15). Another research has done on inhibitory activity of 7 red macroalgae on monophenolase activity of mushroom tyrosinase by Heo *et al*. at concentration of 100 μg/mL of algal extracts, which 0.61-8.84% inhibition has reported (32). In our findings, there was no inhibitory effect on monophenolase activity for *D. simplex *at 100 μg/mL, although, it inhibited oxidation of L-Dopa by 28.78% at the same concentration. 

*In-vivo* tests, using animal models or humans is the most physiologically relevant experiments, but most often these assays are expensive and laborious and a large amount of precious compounds are needed to perform. Overall, there is an increasing pressure to limit the use of animals in researches exceptionally, for tests of preclinical toxicity and safety assessments (33). Zebrafish (Danio rerio) belongs to tropical fresh waters. Its small size, transparent body, being easy to collect a large number of embroys, low cost, rapid embryogenesis, and physiological similarities to mammals are the reasons to be selected as a very useful vertebrate model. Zebrafish are used in the fields of molecular genetics and developmental biology. Recently, it has been used as a model for drug discovery and toxicology studies. Fish embryos absorb molecules through the skin and gills in the early hours after fertilization, but seven days after fertilization molecules are absorbed through the mouth rather than the skin. Since the zebrafish has melanin pigment on the surface of the body, it is a suitable model for melanogenesis observation without using complex laboratory processes (15). Thus, zebrafish was selected for *in-vivo* study of anti-melanogenesis effects of red macroalgae. 

*D. simplex* and kojic acid inhibited tyrosinase activity of zebrafish by 43.18%, and 50.45%, respectively. *D. simplex* extract showed anti-melanogenesis activity comparable to kojic acid as the depletion of total amount of melanin were 47.2% and 50.21%, for *D. simplex* and kojic acid respectively (Figure 1). In a similar study on zebrafish model, two brown algae, *Ecklonia cava,* and *Sargassum silquastrum* inhibited tyrosinase activity by 48% and 50%, and reduced total melanin content by 43% and 50%, respectively at concentration of 100 μg/mL (15). In our study, anti-tyrosinase and anti-melanogenesis effects of *D. simplex *are comparable to those values of these brown seaweeds. *D. simplex* inhibited zebrafish tyrosinase rather than mushroom one, while; kojic acid reduced mushroom tyrosinase activity more than zebrafish tyrosinase (Figure 2). This indicates that the source of tyrosinase is the main factor of tyrosinase inhibition studies. In addition, other factors such as the ability of penetration into the skin are important for *in-vivo *studies because they can interfere in tyrosinase inhibitory results. 

## Conclusion

This study documented anti-melanogenesis of red macroalgae including, *D. simplex*, *L. pappilosa* and *L. paniculata* using *in-vitro* and *in-vivo* models. *D. simplex* exhibited the most inhibitory effect on mushroom tyrosinase as compared to the other tested seaweeds and inhibited melanogenesis and tyrosinase activity of zebrafish comparable to kojic acid. Based on our findings, *D. simplex*, can be considered as a high potential whitening agent for using in cosmetics and medicinal formulations to prevent and treat hyperpigmentation diseases. More studies needed to be done on the other mechanisms of melanogenesis inhibition and isolation and identification of their compounds and activity of isolated compounds with different *in-vitro* and *in-vivo* mechanisms to prove an association between the biological active compounds of *L. papillosa* and *D. simplex* and anti-melanogenesis mechanisms. 
